# Effect of Hypertension Comorbidity on Clinical Characteristics of COVID-19 Patients Infected by the Wild-Type, the Delta or Omicron Variant SARS-CoV-2

**DOI:** 10.31083/j.rcm2312395

**Published:** 2022-12-02

**Authors:** Jinhui Zhang, Jianguo Zhang, Zhimin Tao

**Affiliations:** ^1^Department of Emergency Medicine, The Affiliated Hospital, Jiangsu University, 212001 Zhenjiang, Jiangsu, China; ^2^Department of Critical Care Medicine, The Affiliated Hospital, Jiangsu University, 212001 Zhenjiang, Jiangsu, China; ^3^Jiangsu Province Key Laboratory of Medical Science and Laboratory Medicine, School of Medicine, Department of Laboratory Medicine, Jiangsu University, 212013 Zhenjiang, Jiangsu, China

**Keywords:** SARS-CoV-2, COVID-19, hypertension, omicron variant

## Abstract

**Background:**

Hypertension was the most common comorbidity in patients with the coronavirus disease 2019 (COVID-19). We aim to study the effect of comorbid hypertension on the clinical characteristics of COVID-19 patients with the underlying mechanism.

**Methods:**

We retrospectively analyzed 459, 336 and 659 COVID-19 patients who were infected by the wild-type, the delta and omicron variant, respectively, including their demographic information, medical history, immunization record (if available), and laboratory parameters, to investigate the clinical differences between COVID-19 patients with and without hypertension.

**Results:**

In this study 26.1%, 26.8%, and 12.9% of COVID-19 patients had pre-existing hypertension in the cohort of wild-type, delta, and omicron variant, respectively. Compared to non-hypertensive peers, hypertension patients demonstrated older age, higher occurrence of other major comorbidities, and poorer blood or coagulation parameters, showing worse prognosis. In case of the delta or omicron variant of severe acute respiratory syndrome coronavirus 2 (SARS-CoV-2) infection, hypertension patients produced robust antibody responses, although indistinguishable whether it was due to vaccination or natural infection and resembled those of non-hypertensive peers in blood cell and coagulation profiles with still varying viremic damages to major organs.

**Conclusions:**

Resultantly, COVID-19 infection promoted pro-inflammatory and pro-thrombotic states in hypertension patients, whereas vaccinated individuals would exhibit favorable prognoses.

## 1. Introduction

A novel viral pneumonia broke out in December 2019 and developed into a global health emergency. The responsible pathogen was named as the severe acute respiratory syndrome coronavirus 2 (SARS-CoV-2) and identified as a positive-sense single-stranded RNA virus and the seventh member of the coronavirus family that infects human [1, 2]. The induced coronavirus disease 2019 (COVID-19) was later declared a pandemic by the World Health Organization [3]. Amid the pandemic, waves of new SARS-CoV-2 variants incessantly surged, rapidly spread, and ruthlessly hit the COVID-19-weary world, which substantially impacted the socioeconomic sectors [4]. This viremia poses a serious threat to human health, with particularly increased risk for those with weakened immune system [5]. As of June 12, 2022, the cumulative number of infection reached over 533 million with a death toll exceeding 6 million, indicating a fatality rate of ~1.2% [6].

The typical clinical manifestations of COVID-19 are flu-like, including fever, cough, chest pain, and dyspnea [7, 8]. While most patients exhibit mild-to-moderate symptoms, some patients’ conditions may rapidly deteriorate or even become life-threatening. The consensus is reached that among various risk factors, increasing age and specific comorbidities play a crucial role in COVID-19 morbidity and mortality [9, 10]. Since the ratio of people with at least one underlying medical condition in the entire population rises with age, the comorbidity constitutes a significant and independent risk factor for worsened prognoses of COVID-19 patients [11].

During the COVID-19 outbreak in China in early 2020, hypertension was identified as the leading comorbidity with SARS-CoV-2 infection. The percentage of hypertension patients was higher in the severe or death group than in the non-severe or survival group [12, 13, 14, 15, 16, 17]. Due to the varying sizes of different study cohorts, the ratio of COVID-19 patients with co-existing hypertension ranges from 15% to 34%, while hypertension as an independent risk factor of COVID-19 severity is considered the predominant comorbidity [18, 19, 20]. Nevertheless, the exact role of the underlying hypertensive disease in the development of COVID-19 remains poorly studied and understood. The influence of hypertension on the patients infected by new variants of SARS-CoV-2 remains unclear.

In this study, we first compared the clinical characteristics between COVID-19 patients in an intensive care unit (ICU) and a non-ICU, determining variables associated with disease severity and mortality. We also examined the differences between COVID-19 patients with and without hypertension, infected by either the wild-type or the delta or omicron variant SARS-CoV-2, with a further exploration of how this specific comorbidity adversely affected the disease progression in COVID-19 patients.

## 2. Methods

### 2.1 Patients

459 COVID-19 patients were admitted at the First People’s Hospital of Jiangxia District (FPHJD) in Wuhan and the Huangshi City Hospital (HCH), Hubei, China, from January to April 2020, where 206 developed into severe cases and were transferred to ICU, and 253 stayed in non-ICU isolation ward. The COVID-19 severity was defined according to the management guideline by China National Health Commission [21]. Briefly, adult severe cases were typically presented as respiratory distress ≥30 breaths/min, or oxygen saturation ≤93% at rest, or arterial partial pressure of oxygen/fraction of inspired oxygen less than or equal to 300 mmHg. The severity rate was calculated as the portion of severe patients among all the COVID-19 patients included. Besides, 336 and 659 mild COVID-19 patients respectively infected by the delta and omicron variant of SARS-CoV-2 were included, hospitalized at the Third People’s Hospital of Yangzhou City (TPHYC) in August 2021 and the Fifth People’s Hospital of Suzhou (TFPHS, the Affiliated Infectious Diseases Hospital of Soochow University) in March 2022, respectively. Among them, no patients were reported to develop severity or mortality. All COVID-19 patients were confirmed as previously reported [8, 22, 23]. We excluded patients with malignancy, pregnancy, or immunodeficiency, patients younger than 18 years, and patients who failed to complete blood examinations. The study was approved by the Research Ethics Commission of FPHJD, HCH, TPHYC and TFPHS, respectively. The study was also reviewed and approved by the Research Ethics Commission of the Affiliated Hospital of Jiangsu University, with which all authors were affiliated. The patient information remained anonymous, and the requirement for written informed consent was waived due to the emergency situation of COVID-19. Patients were defined as having hypertension based on previous diagnoses with a systolic blood pressure of ≥140 mmHg and/or a diastolic blood pressure of ≥90 mmHg [24], or current use of antihypertensive medication. Cardiovascular diseases refer to a series of diseases involving the circulatory system, including coronary artery disease, cerebrovascular disease, peripheral artery disease and aortic atherosclerosis [25]. Bronchitis is a non-specific inflammation in the trachea and bronchial mucosa and surrounding tissues, one kind of chronic obstructive pulmonary disease (COPD) [26].

### 2.2 Procedures

COVID-19 patients were hospitalized and treated as described previously [22, 23]. For hypertension patients with COVID-19, specified patient management and therapeutical strategies were directed and administered clinically [27]. Blood analyses of patients were conducted as previously reported [8, 22, 23].

### 2.3 Vaccinations

Inactivated vaccines were administered to the delta or omicron COVID-19 patients who were admitted in TPHYC and TFPHS, respectively, and the serological tests of patients based on detection of SARS-CoV-2-specific immunoglobulin M (IgM) and immunoglobulin G (IgG) were conducted as reported [22, 23].

### 2.4 Statistical Analysis

Data were summarized as the median and interquartile range values for continuous variables and frequencies for categorical variables. For comparisons between two groups, the Mann-Whitney U test was used for continuous variables. Categorical variables were examined using the Chi-Square test. The selected variables according to their clinical relevance and statistical significance in univariate analysis (*p <* 0.05) were further assessed by multivariate logistic regression analyses, to explore the independent risk factors associated with different group pairings. Survival curves were plotted using the Kaplan-Meier method and compared between patients with and without hypertension using the log-rank test. All *p* values were two-sided, and *p* values < 0.05 were considered statistically significant. All statistical analyses were performed using SPSS version 16.0 (SPSS Inc., Chicago, IL, USA), following the methods as reported [22, 23].

## 3. Results

### 3.1 Clinical Characteristics of COVID-19 Patients with Different Outcomes following Hospitalization

We first grouped the COVID-19 patients into non-ICU (mild) and ICU (severe) groups, whom we examined with regards to their baseline characteristics. Compared to the non-ICU group, the ICU group had much higher median age and male:female ratio, and higher incident of major comorbidities (**Supplementary Table 1**). In addition, ICU patients demonstrated significantly worse blood profile and more severe coagulopathy, suggestive of increased risk for viral hits to impair the major organs. Variables with clinical relevance and significant difference (*p <* 0.05) in univariate analyses between non-ICU and ICU groups were further performed using multivariate logistic regression analysis to identify the independent risk factors associated with the severity of COVID-19. The results in **Supplementary Table 2** show that age, hypertension, red blood cells, D-dimer, ALT, BUN, and potassium levels predict the severity of COVID-19. Severe patients later transferred to the ICU were regrouped into survived and deceased groups, based on the final disease outcome, and their baseline characteristics were compared, showing worsened conditions in the deceased group. Variables with clinical relevance and significant difference (*p <* 0.05) in univariate analysis between the survived and deceased groups were performed using multivariate logistic regression analysis (**Supplementary Table 3**). Hypertension as a comorbidity was identified as an independent risk factor for COVID-19 mortality.

### 3.2 Differences between the Clinical Characteristics of COVID-19 Patients with or without Hypertension when Infected by the Wild-Type SARS-CoV-2

We next regrouped all 459 patients infected by the wild-type SARS-CoV-2 into one group with hypertension and the other without hypertension and compared their clinical manifestations. Results are shown in Table 1. Evidently, compared to the non-hypertensive group, the hypertensive group showed much higher patient ages but similar male:female gender ratio. Although both groups had similar frequencies of diabetes and bronchitis, the hypertensive group showed higher occurrence of cardiovascular comorbidity.

**Table 1.** **Comparison of clinical characteristics between COVID-19 patients with and without hypertension, infected by the wild-type SARS-CoV-2**.

			Hypertension (n = 120)	Non–hypertension (n = 339)	*p*
Age, years			67.0 (57.3–75.0)	55.0 (42.0–68.0)	<0.001
Male, N (n%)			67 (55.8)	182 (53.7)	0.685
Comorbidity					
	Diabetes		24 (20.0)	46 (13.6)	0.092
	Cardiovascular diseases		22 (18.3)	29 (8.6)	0.003
	Bronchitis		9 (7.5)	25 (7.4)	0.964
Blood cell count		Normal range			
	WBCs, × ${\mathrm{10}}^{9}$/L	3.5–9.5	7.3 (5.4–9.7)	6.2 (4.8–8.3)	0.008
	Neutrophils, × ${\mathrm{10}}^{9}$/L	1.8–6.3	5.6 (3.8–7.6)	4.5 (2.9–6.7)	0.003
	Lymphocytes, × ${\mathrm{10}}^{9}$/L	1.1–3.2	1.0 (0.6–1.3)	1.0 (0.7–1.4)	0.101
	Monocytes, × ${\mathrm{10}}^{9}$/L	0.1–0.6	0.5 (0.3–0.6)	0.4 (0.3–0.6)	0.517
	RBCs, × ${\mathrm{10}}^{12}$/L	3.8–5.1	3.8 (3.2–4.3)	4.1 (3.5–4.5)	0.002
	Hemoglobin, g/L	115–150	112 (91–131)	122 (106–137)	0.001
	HCT, %	35–50	34.1 (28.4–38.4)	36.6 (32.0–40.2)	0.001
	Platelets, × ${\mathrm{10}}^{9}$/L	125–350	175 (117–268)	193 (146–260)	0.264
	MPV, fL	7.4–12.5	10.8 (10.2–11.7)	10.7 (9.9–11.4)	0.164
Coagulation factor					
	Prothrombin time, s	9–13	13.3 (12.3–14.5)	13.3 (12.3–14.1)	0.659
	INR	0.8–1.2	1.1 (1.0–1.2)	1.1 (1.0–1.2)	0.832
	aPTT, s	23.3–32.5	31.0 (28.5–34.9)	30.2 (28.0–32.4)	0.061
	Thrombin time, s	14–21	16.7 (15.7–18.0)	16.3 (15.3–17.4)	0.071
	Fibrinogen, g/L	2–4	4.1 (3.3–5.3)	3.7 (2.7–4.6)	0.005
	D-dimer, mg/L	<0.55	1.4 (0.5–3.9)	0.97 (0.3–2.8)	0.052
Metabolic panel					
	CRP, mg/L	0–10	27.8 (12.5–64.2)	25.0 (12.8–59.2)	0.744
	PCT, ng/mL	<0.1	0.8 (0.4–1.6)	1.1 (0.4–1.7)	0.402
	Total bilirubin, μmol/L	3–22	17.4 (13.3–28.5)	17.1 (12.1–27.0)	0.591
	Direct bilirubin, μmol/L	0–5	8.1 (4.4–13.8)	7.4 (4.0–13.3)	0.427
	Indirect bilirubin, μmol/L	0–19	10.5 (6.6–14.8)	10.0 (6.5–14.6)	0.997
	ALT, U/L	9–50	34.6 (22.5–46.1)	31.0 (19.7–40.5)	0.033
	AST, U/L	15–40	33.2 (21.7–49.8)	32.4 (17.9–44.9)	0.122
	ALP, U/L	32–126	67.0 (50.0–84.0)	70.0 (52.0–96.5)	0.224
	GGT, U/L	12–73	53.0 (30.5–82.6)	43.0 (26.0–68.0)	0.010
	Total protein, g/L	63–82	59.5 (52.8–65.2)	58.1 (52.0–64.4)	0.321
	Albumin, g/L	35–50	32.6 (29.3–36.8)	33.5 (29.5–37.6)	0.338
	Globulin, g/L	20–30	25.0 (20.3–30.1)	24.4 (19.8–28.6)	0.114
	ADA, U/L	4–22	14.1 (11.4–18.7)	14.1 (10.9–17.9)	0.669
	BUN, mmol/L	2.86–8.2	5.5 (4.0–10.6)	5.0 (3.8–8.3)	0.058
	Creatinine, μmol/L	31.7–133	67.3 (52.6–83.1)	64.5 (51.8–78.3)	0.236
	Glucose, mmol/L	3.89–6.11	8.7 (6.4–12.8)	8.7 (6.3–12.7)	0.967
	LDH, U/L	80–285	328.0 (208.5–489.5)	365.0 (226.4–536.0)	0.349
	CPK, U/L	38–174	72.5 (48.0–117.5)	64.00 (48.0–103.0)	0.237
	CK-MB, U/L	0–25	45.9 (23.3–80.4)	44.7 (25.3–70.2)	0.799
	Potassium, mmol/L	3.5–5.3	4.0 (3.5–4.4)	4.2 (3.6–4.5)	0.191
	Sodium, mmol/L	137–147	141.8 (137.2–147.4)	142.5 (137.4–146.6)	0.805
Outcome					
	Severity rate (%)		73 (60.8)	133 (39.2)	<0.001
	Mortality rate (%)		42 (35.0)	54 (15.9)	<0.001

Abbreviations: WBC, white blood cell; RBC, red blood cell; HCT, hematocrit; MPV, mean platelet volume; INR, international normalized ratio; aPTT, activated partial thromboplastin time; CRP, c-reactive protein; PCT, procalcitonin; ALT, alanine aminotransferase; AST, aspartate aminotransferase; ALP, alkaline phosphatase; GGT, γ-glutamyl transferase; ADA, adenosine deaminase; BUN, blood urea nitrogen; LDH, lactate dehydrogenase; CPK, creatine phosphokinase; CK-MB, creatine kinase isoenzyme.

In hematological analysis, the hypertensive group revealed more blood cell abnormalities such as leukocytosis, neutrophilia, and anemia, but the levels of abnormalities like lymphocytopenia and thrombocytopenia were similar to those in the non-hypertensive group. Notably, most coagulation factors (except for the fibrinogen level) or metabolic biomarkers (except for ALT and γ-glutamyl transferase, or GGT) did not show any substantial difference between the two groups, implying that hypertension may not deteriorate the hematological indices or coagulation profiles when patients were infected with SARS-CoV-2.

**Fig. 1.**
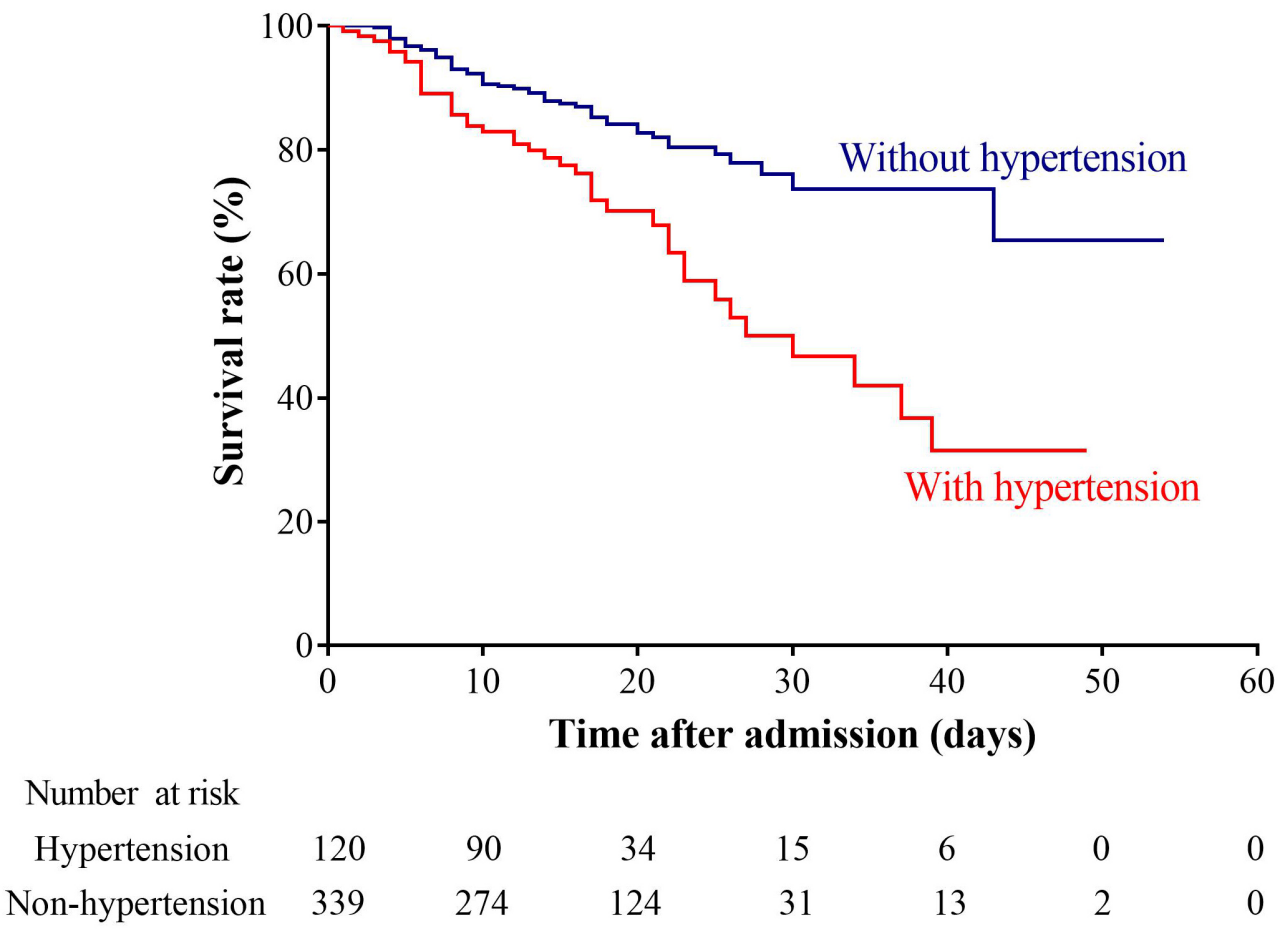
**Kaplan-Meier survival curve for COVID-19 patients infected by the wild-type SARS-CoV-2 with or without hypertension**.

However, consequently, COVID-19 patients with hypertension versus those without hypertension had a severity rate of 60.8% versus 39.2% (*p *< 0.001), and a mortality rate of 35.0% versus 15.9% (*p *< 0.001), respectively. The Kaplan-Meier survival curve demonstrated a clear trend toward poorer survival in the hypertensive COVID-19 group compared to that in the non-hypertensive group, with statistical significance (*p *< 0.001) (Fig. 1). Therefore, hypertension serves a prognostic indicator for both severity and mortality of COVID-19 patients infected by the wild-type SARS-CoV-2.

### 3.3 Differences between the Clinical Characteristics of COVID-19 Patients with or without Hypertension when Infected by the Delta Variant SARS-CoV-2 

We next grouped 336 patients infected by the delta variant SARS-CoV-2 into one with hypertension and the other without hypertension and compared their clinical manifestations. Results are shown in Table 2. Compared to the non-hypertensive group, the hypertensive group showed much higher age but similar male:female sex ratio and possessed similar frequencies of cardiovascular diseases and bronchitis, but a higher occurrence of diabetes.

**Table 2.** **Comparison of clinical characteristics between COVID-19 patients with and without hypertension, infected by the delta variant SARS-CoV-2**.

			Hypertension (n = 90)	Non–hypertension (n = 246)	*p*
Age, years			65.0 (54.8–73.3)	44.0 (31.0–60.3)	<0.001
Male, N (n%)			48 (53.3)	143 (58.1)	0.432
Comorbidity					
	Diabetes		22 (24.4)	9 (3.6)	<0.001
	Cardiovascular diseases		11 (12.2)	11 (4.5)	0.229
	Bronchitis		3 (3.3)	1 (0.4)	0.105
Vaccination times					
	0		38 (42.2)	81 (32.9)	0.115
	1		21 (23.3)	40 (16.3)	0.136
	2		31 (34.4)	125 (50.8)	0.008
Antibody response					
None			51 (56.7)	111 (45.1)	0.061
	IgG		37 (41.1)	133 (54.1)	0.035
	IgM		21 (23.3)	51 (20.7)	0.607
	IgG+IgM		19 (21.1)	49 (19.9)	0.810
Blood cell count		Normal range			
	WBCs, × ${\mathrm{10}}^{9}$/L	3.5–9.5	5.5 (4.3–6.7)	5.0 (3.9–6.2)	0.009
	Neutrophils, × ${\mathrm{10}}^{9}$/L	1.8–6.3	3.6 (3.7–4.7)	3.2 (2.3–4.2)	0.015
	Lymphocytes, × ${\mathrm{10}}^{9}$/L	1.1–3.2	1.1 (0.8–1.5)	1.1 (0.8–1.5)	0.834
	Monocytes, × ${\mathrm{10}}^{9}$/L	0.1–0.6	0.5 (0.4–0.7)	0.5 (0.4–0.6)	0.171
	RBCs, × ${\mathrm{10}}^{12}$/L	3.8–5.1	4.5 (4.1–4.9)	4.5 (4.2–4.9)	0.618
	Hemoglobin, g/L	115–150	136.0 (124.0–150.0)	137.0 (124.0–148.0)	0.860
	HCT, %	35–45	39.6 (36.4–43.4)	39.8 (36.8–42.8)	0.827
	Platelets, × ${\mathrm{10}}^{9}$/L	125–350	159.5 (134.8–199.0)	172.0 (132.0–213.3)	0.179
	MPV, fL	9–13	11.0 (10.5–11.8)	11.1 (10.5–11.9)	0.600
Coagulation factor					
	Prothrombin time, s	9–15	11.8 (11.4–12.2)	12.1 (11.5–12.6)	0.001
	INR	0.8–1.2	1.0 (1.0–1.1)	1.1 (1.0–1.1)	0.002
	aPTT, s	20–40	29.1 (27.0–32.2)	30.5 (27.6–32.9)	0.180
	Thrombin time, s	14–21	17.9 (17.6–18.7)	17.9 (17.2–18.5)	0.150
	Fibrinogen, g/L	2–4	3.5 (2.9–4.0)	3.2 (2.7–3.9)	0.045
	D-dimer, mg/L	<0.55	0.4 (0.2–0.8)	0.4 (0.2–0.5)	0.115
Metabolic panel					
	CRP, mg/L	0–3	16.3 (4.8–34.6)	11.5 (3.9–26.4)	0.017
	PCT, ng/mL	<0.5	0.0 (0.0–0.1)	0.0 (0.0–0.1)	0.002
	Total bilirubin, μmol/L	3–22	9.0 (6.6–13.1)	8.1 (5.9–10.6)	0.089
	Direct bilirubin, μmol/L	0–5	4.2 (3.1–5.7)	3.8 (2.9–4.7)	0.014
	Indirect bilirubin, μmol/L	0–19	4.7 (3.3–6.4)	4.3 (2.9–6.2)	0.286
	ALT, U/L	7–40	24.0 (16.1–37.4)	16.0 (11.0–27.1)	<0.001
	AST, U/L	13–35	26.7 (20.7–38.1)	20.7 (16.6–28.0)	<0.001
	ALP, U/L	35–100	87.0 (72.0–107.3)	76.0 (66.0–93.0)	<0.001
	GGT, U/L	7–45	31.5 (19.0–59.3)	21.0 (13.0–36.0)	<0.001
	Total protein, g/L	63–85	72.5 (68.3–77.6)	72.5 (68.7–76.8)	0.980
	Albumin, g/L	35–50	45.3 (42.5–48.2)	46.9 (44.0–49.4)	0.012
	Globulin, g/L	20–40	27.3 (24.0–30.1)	26.4 (23.4–28.9)	0.048
	ADA, U/L	10–15	14.0 (12.0–17.0)	13.0 (11.0–16.0)	0.026
	BUN, mmol/L	2.7–7.5	5.1 (4.4–6.5)	4.2 (3.4–5.1)	<0.001
	Creatinine, μmol/L	41–73	77.0 (64.8–93.3)	69.0 (59.0–83.0)	<0.001
	Glucose, mmol/L	3.89–6.11	6.7 (5.5–9.2)	5.7 (4.8–7.2)	<0.001
	LDH, U/L	120–250	204.5 (185.0–251.3)	193.5 (167.0–232.5)	0.002
	CPK, U/L	40–200	100.0 (63.0–193.3)	84.0 (57.8–120.3)	0.008
	CK-MB, U/L	0–25	13.5 (10.6–16.8)	12.8 (10.1–15.9)	0.238
	Potassium, mmol/L	3.5–5.3	3.5 (3.2–3.8)	3.7 (3.4–4.0)	0.005
	Sodium, mmol/L	137–147	137.5 (135.0–139.0)	138.0 (136.0–139.0)	0.485

The two groups had similar ratios of unvaccinated and partially vaccinated (single-dose) patients, but the hypertensive group had fewer patients who were fully vaccinated (two-dose). Concurrently, all COVID-19 patients upon admission were tested for antibody production in the sera upon hospitalization, although it was not possible to distinguish whether these antibody responses had resulted from natural exposure or recent vaccination. The patient statuses regarding the presence of no antibody, only IgM, or IgG+IgM production between the two groups were similar, whereas IgG detection in the hypertensive group was not common.

In the laboratory data of blood tests, the hypertensive group displayed more severe leukocytosis and neutrophilia, but similar lymphocytopenia, monocytosis, anemia, and thrombocytopenia to those in the non-hypertensive group. While most coagulation factors did not reflect a worsened condition in the hypertensive group, many of their metabolic biomarkers mirrored substantially deteriorated conditions, exemplified by heightened levels of CRP, PCT, direct bilirubin, ALT, AST, BUN, creatinine, glucose, lactate dehydrogenase (LDH), CPK, and potassium.

### 3.4 Differences between the Clinical Characteristics of COVID-19 Patients with or without Hypertension when Infected by the Omicron Variant SARS-CoV-2 

We then grouped 659 patients infected by the omicron variant SARS-CoV-2 into one with hypertension and the other without hypertension and compared their clinical characteristics. Results are shown in Table 3. Compared to the non-hypertensive group, the hypertensive group exhibited much higher age but similar male:female ratio and owned higher occurrence of diabetes, cardiovascular diseases and bronchitis.

**Table 3.** **Comparison of clinical characteristics between COVID-19 patients with and without hypertension, infected by the omicron variant SARS-CoV-2**.

			Hypertension (n = 85)	Non–hypertension (n = 574)	*p*
Age, years			61.0 (50.0–69.0)	36.0 (30.0–48.0)	<0.001
Male, N (n%)			47 (55.3)	302 (52.6)	0.644
Comorbidity					
	Diabetes		14 (16.5)	12 (2.1)	<0.001
	Cardiovascular diseases		3 (3.5)	4 (0.7)	0.049
	Bronchitis		4 (4.7)	4 (0.7)	0.009
Vaccination times					
	0		17 (20.0)	60 (10.5)	0.011
	1		4 (4.7)	43 (7.5)	0.352
	2		35 (41.2)	287 (50.0)	0.129
	3		29 (34.1)	184 (32.1)	0.704
Antibody response					
	None		45 (53.0)	329 (57.3)	0.447
	IgG		38 (44.7)	244 (42.5)	0.702
	IgM		0 (0)	0 (0)	—
	IgG+IgM		2 (2.4)	1 (0.2)	0.045
Blood cell count		Normal range			
	WBCs, × ${\mathrm{10}}^{9}$/L	3.5–9.5	6.5 (5.3–8.0)	6.1 (4.9–7.6)	0.123
	Neutrophils, × ${\mathrm{10}}^{9}$/L	1.8–6.3	4.8 (3.6–6.2)	4.5 (3.2–5.9)	0.230
	Lymphocytes, × ${\mathrm{10}}^{9}$/L	1.1–3.2	0.8 (0.6–1.3)	0.9 (0.6–1.3)	0.411
	Monocytes, × ${\mathrm{10}}^{9}$/L	0.1–0.6	0.6 (0.4–0.8)	0.5 (0.4–0.7)	0.020
	RBCs, × ${\mathrm{10}}^{12}$/L	4.3–5.8	4.7 (4.4–5.0)	4.8 (4.4–5.3)	0.043
	Hemoglobin, g/L	130–175	141.0 (129.5–149.0)	142.5 (131.0–155.0)	0.167
	HCT, %	40–50	42.0 (39.1–44.4)	42.3 (38.9–46.0)	0.214
	Platelets, × ${\mathrm{10}}^{9}$/L	125–350	196.0 (169.5–241.5)	213.0 (179.0–249.0)	0.138
	MPV, fL	9–13	10.1 (9.2–11.0)	10.1 (9.4–10.9)	0.697
Coagulation factor					
	Prothrombin time, s	10–14	11.3 (10.6–12.2)	11.4 (10.6–12.5)	0.424
	INR	0.8–1.2	0.9 (0.9–1.0)	1.0 (0.9–1.0)	0.833
	aPTT, s	20–40	29.1 (25.8–33.2)	29.2 (25.7–33.1)	0.970
	Thrombin time, s	14–21	18.5 (15.8–19.3)	18.4 (15.1–19.4)	0.640
	Fibrinogen, g/L	2–4	2.8 (2.5–3.4)	2.7 (2.2–3.3)	0.194
	D-dimer, mg/L	<0.55	0.2 (0.2–0.5)	0.2 (0.2–0.4)	0.260
Metabolic panel					
	CRP, mg/L	0–10	3.7 (1.6–9.4)	3.5 (1.0–8.5)	0.499
	PCT, ng/mL	<0.5	0.1 (0.1–0.2)	0.1 (0.1–0.2)	0.676
	Total bilirubin, μmol/L	3–22	72.7 (67.3–76.9)	73.3 (69.2–77.9)	0.336
	Direct bilirubin, μmol/L	0–5	2.6 (1.2–3.7)	2.6 (1.0–3.7)	0.635
	Indirect bilirubin, μmol/L	0–19	8.2 (4.5–11.4)	6.8 (4.1–9.9)	0.055
	ALT, U/L	21–72	29.0 (24.5–36.0)	29.0 (22.0–40.0)	0.820
	AST, U/L	17–59	26.0 (22.0–33.0)	24.0 (20.0–30.0)	0.057
	ALP, U/L	38–126	82.0 (68.0–98.5)	68.5 (57.0–82.0)	<0.001
	GGT, U/L	15–73	22.0 (17.0–34.5)	20.0 (14.0–30.0)	0.012
	Total protein, g/L	63–82	72.7 (67.3–76.9)	73.3 (69.2–77.9)	0.336
	Albumin, g/L	35–50	44.9 (42.5–47.4)	45.6 (43.2–47.9)	0.159
	Globulin, g/L	20–30	27.3 (24.6–30.6)	27.2 (24.2–30.9)	0.738
	BUN, mmol/L	3.2–7.1	5.5 (4.3–6.7)	4.3 (3.5–5.1)	<0.001
	Creatinine, μmol/L	58–110	67.2 (51.5–80.7)	58.2 (46.8–69.6)	<0.001
	Glucose, mmol/L	4.10–5.90	6.3 (5.6–7.3)	5.9 (5.3–6.7)	0.010
	LDH, U/L	120–246	205.0 (184.0–239.0)	193.5 (172.0–225.0)	0.021
	CPK, U/L	55–170	72.0 (49.0–131.5)	71.5 (50.0–105.0)	0.463
	Potassium, mmol/L	3.50–5.01	3.9 (3.6–4.1)	3.9 (3.7–4.2)	0.111
	Sodium, mmol/L	137–145	139.0 (136.1–141.5)	139.1 (135.7–141.6)	0.510

Both groups had similar ratios of partially, fully and booster vaccinated patients, but the hypertensive group had a much higher ratio of unvaccinated patients. The antibody responses in both groups of patients showed similarity in producing no antibody and only IgG, while all patients had no IgM production and extremely low co-production of IgG+IgM.

In the laboratory tests, the two groups demonstrated similar degrees of leukocytosis, neutrophilia, lymphocytopenia, and thrombocytopenia, although monocytosis and anemia were more severe in the hypertensive group. All coagulation factors showed similar conditions between the hypertensive and non-hypertensive groups. Most metabolic biomarkers did not differentiate one group from the other, except that the hypertension patients had more elevated levels of ALP, GGT, BUN, creatinine, glucose, and LDH.

Finally, we conducted a direct comparison by listing all parameters with significant differences (*p <* 0.05) between hypertensive and non-hypertensive groups with either the wild-type or the delta or omicron variant SARS-CoV-2 infection (Table 4). The difference between normotensive and hypertension patients with delta variant infection exhibited greater diversity than that with wild-type or omicron variant SARS-CoV-2 infection.

**Table 4.** **The baseline clinical characteristics with significant differences (*p <* 0.05) between hypertensive and non-hypertensive groups in the wild-type or the delta or omicron variant SARS-CoV-2 infections were listed and compared**.

**Differences between hypertensive and non-hypertensive groups**					
**Wild type**	***p***	**Delta variant**	***p***	**Omicron variant**	***p***
Age	<0.001	Age	<0.001	Age	<0.001
**Comorbidity**		**Comorbidity**			
		Diabetes	<0.001	Diabetes	<0.001
Cardiovascular diseases	0.003			Cardiovascular diseases	0.049
				Bronchitis	0.009
**Blood cell count**		**Blood cell count**		**Blood cell count**	
WBCs	0.008	WBCs	0.009		
Neutrophils	0.003	Neutrophils	0.015		
				Monocytes	0.020
RBCs	0.002			RBCs	0.043
Hemoglobin	0.001				
HCT	0.001				
**Coagulation factor**		**Coagulation factor**		**Coagulation factor**	
		Prothrombin time	0.001		
		INR	0.002		
Fibrinogen	0.005	Fibrinogen	0.045		
**Metabolic panel**		**Metabolic panel**		**Metabolic panel**	
		CRP	0.017		
		PCT	0.002		
		Direct bilirubin	0.014		
ALT	0.033	ALT	<0.001		
		AST	<0.001		
		ALP	<0.001	ALP	<0.001
GGT	0.010	GGT	<0.001	GGT	0.012
		Albumin	0.012		
		Globulin	0.048		
		ADA	0.026		
		BUN	<0.001	BUN	<0.001
		Creatinine	<0.001	Creatinine	<0.001
		Glucose	<0.001	Glucose	0.010
		LDH	0.002	LDH	0.021
		CPK	0.008		
		Potassium	0.005		

## 4. Discussion

Our current report agrees that in infections of SARS-CoV-2 and its delta or omicron variant, patients with the pre-existing hypertension were associated with more severe abnormalities in the blood cell count, platelet function, coagulation profile and/or metabolic biomarkers, leading to higher severity and mortality of COVID-19 patients. Our results also reveal that differences in clinical characteristics between normotensive and hypertension patients infected by the delta variant of SARS-CoV-2 are more diverse than those in patients with the wild-type or omicron variant infection, although infection by the two variants leads to significantly reduced severity and fatality.

Since the onset of the pandemic, a flurry of research on COVID-19 has mushroomed to help understand this devastating disease. Age and comorbidities in COVID-19 patients contribute to their severity and mortality [28]. Typically, these comorbidities comprise hypertension, diabetes, and cardiovascular diseases, among which hypertension is predominant [29, 30, 31]. In fact, hypertension has been listed as one of the most common comorbidities in patients with other coronavirus (CoV) infections, such as those with severe acute respiratory syndrome CoV, and Middle East respiratory syndrome CoV, and human CoV 229E [32, 33].

The pathological origin of hypertension can be multifactorial, including genetic predisposition and acquired lifestyle [34]. Both prevalence and severity of hypertension climb as the patient’s age increases. In the United States, ~60% of the population has hypertension by the age of 60, and the lifetime risk of developing hypertension is >90% for those aged 55–65 [35]. In China, 53.2% of the population aged above 60 years is hypertensive [36]. The ratio of hypertension patients in the general population is expected to further increase as the global society trends toward aging. In fact, aging is an important factor that puts the COVID-19 patients at elevated risks for rapidly clinical deterioration, due to the associated immunosenescence and comorbid disorder that aggravate the pro-inflammatory states [37, 38].

Simultaneously, the top comorbidities in hypertension patients include coronary heart disease, diabetes, hyperlipidemia, and arteriosclerosis [39]. Our current findings stand in agreement with those reports. In all cohorts, hypertension patients showed much higher age than non-hypertensive, while diabetes and cardiovascular diseases were two leading comorbidities within hypertensive COVID-19 patients. Moreover, the male predisposition to COVID-19 severity has been attributed to unfavorable socioeconomic factors (prone to hygiene reluctancy and social gathering) and sex-specific immune responses (due to male-exclusive hormones) [40]. Research on COVID-19 patients with comorbid hypertension and diabetic mellitus indicated that compared to female patients, male patients had a higher proportion of cardiopathy ischemic and lung diseases but a lower proportion of kidney diseases, in association with worse clinical outcomes that include the longer hospital stays and the higher ICU admission and death rate [41].

Pre-existing medical conditions may increase the risk of COVID-19 infectivity, severity, and mortality via two approaches: the first is by enhancing the viral entry of SARS-CoV-2 and the second is by intensifying the viremic effect after the infection has occurred. SARS-CoV-2 employs human angiotensin-converting enzyme 2 (ACE2) as cell entry receptor, further infecting lung, heart, liver, and other organs, and leading to blood coagulopathy and organ dysfunction [7, 8, 42]. Nevertheless, no connection has been reported so far between the usage of anti-hypertensive medications and increased COVID-19 susceptibility, severity, or mortality [43, 44]. Therefore, hypertension patients on medication may not raise the SARS-CoV-2 infectivity, leaning on another postulation that once an individual is infected, hypertension may aggravate viremic effects in COVID-19 patients.

Hypertension induces hemorheological abnormality, causes endothelial dysfunction, and confers hypercoagulation [45]. Concurrently, elevated thrombogenesis and inflammation have been frequently observed in hypertensive emergencies [46]. Although inflammation may cause the development of a hypertensive state, hypertension stimulates immune cell activation and induces cytokine secretion, thereby promoting a variety of inflammatory events [47, 48]. Thus, hypertension is both pro-inflammatory and pro-thrombotic, contributing to organ damage including stroke, heart injury and renal failure. Previously, we reported longstanding hyperinflammatory response and refractory coagulopathy in COVID-19 patients, possibly driven by platelet activation due to SARS-CoV-2 infection [8]. Similar findings have been confirmed by other researchers [49, 50]. Therefore, hypertension patients, once infected with SARS-CoV-2, may exacerbate the pro-inflammatory and pro-thrombotic states, leading to worsened disease course and outcome.

Hypertension reportedly has no significant effect on antibody production after participants have received full-dose mRNA vaccines [51]. Controversial results also suggest that fully vaccinated hypertensive individuals develop lower antibody levels than those of their normotensive peers due to their impaired immunity [52]. Our results are insufficient to evaluate the efficiency of antibody responses in hypertensive COVID-19 patients if infected or vaccinated. Nevertheless, COVID-19 vaccinations are highly recommended for immunocompromised groups at risk, including the hypertensive population, which could prevent worsened disease outcomes upon infection.

This study had several limitations. First, due to the emergency nature of COVID-19, especially during its regional outbreak, many baseline characteristics of hospitalized patients were unavailable or incomplete in this retrospective study. For instance, the body mass index was missing in all cohorts; otherwise, we might have studied the role of obesity as a comorbidity in COVID-19 patients with a possible linkage to the influence of comorbid hypertension. Similarly, it would be meaningful to analyze the type and duration of antihypertensive medications among COVID-19 patients related to their individual disease outcomes. Second, our study cohort was relatively small, further limiting the number of hypertension patients. Thus, it is challenging to minimize the random errors in the results. Third, we may not evaluate the effect of comorbid hypertension on severity and mortality of COVID-19 patients infected by the delta or omicron variant of SARS-CoV-2, due to the lack of data pertaining to the severe or fatal cases in both infections.

## 5. Conclusion

In conclusion, this retrospective study examined the effects of hypertension as a comorbidity on the clinical manifestations of COVID-19 patients infected by the wild-type or the delta or omicron variant SARS-CoV-2 and identified the implicit causation. Our results corroborate that the hypertension-conferred hyperinflammatory and hypercoagulable states may be intensified upon SARS-CoV-2 infection. This partially explains the prognostic value of hypertension as a comorbidity on COVID-19 severity and mortality for patients infected by the wild-type SARS-CoV-2. Compared to the difference in clinical characteristics between normotensive and hypertension patients infected by the wild-type or the omicron variant SARS-CoV-2, the difference in patients with delta variant infection demonstrated greater diversity, although the two variants of SARS-CoV-2 may be less severe and less fatal.

## Data Availability

On reasonable request, the datasets used for the analyses in the current study are available from the corresponding author(s).

## Abbreviations

WBC, white blood cell; RBC, red blood cell; HCT, hematocrit; MPV, mean platelet volume; INR, international normalized ratio; aPTT, activated partial thromboplastin time; CRP, c-reactive protein; PCT, procalcitonin; ALT, alanine aminotransferase; AST, aspartate aminotransferase; ALP, alkaline phosphatase; GGT, γ-glutamyl transferase; ADA, adenosine deaminase; BUN, blood urea nitrogen; LDH, lactate dehydrogenase; CPK, creatine phosphokinase; CK-MB, creatine kinase isoenzyme.

## Supplementary Material

Supplementary material associated with this article can be found, in the online version, at https://doi.org/10.31083/j.rcm2312395.
