# Supplementary material for: Effect of Hypertension Comorbidity on Clinical Characteristics of COVID-19 Patients Infected by the Wild-Type, the Delta or Omicron Variant SARS-CoV-2
Source: Rev Cardiovasc Med. 2022 Dec 2;23(12):395. doi: 10.31083/j.rcm2312395 (PMC11270381; doi:10.31083/j.rcm2312395)
Supplement: Supplementary file 1 [file 2153-8174-23-12-395-s1.docx]

**Supplementary Table 1.** Comparison of clinical characteristics between non-ICU and ICU patients infected with the wild-type SARS-CoV-2, where survived and deceased groups were also compared after ICU transfer.

|  | **Non-ICU (n=253)** | **ICU (n=206)** | ***p*** | **Survived (n=118)** | **Deceased (n=88)** | ***p*** |
| --- | --- | --- | --- | --- | --- | --- |
| Age, years | 52.00 (39.00-63.00) | 66.50 (57.00-76.00) | <0.001 | 63.00 (54.75-76.00) | 68.50 (58.25-77.00) | 0.068 |
| Male, N (n%) | 122 (48.2) | 127 (61.7) | 0.004 | 76 (64.4) | 51 (58.0) | 0.346 |
| **Comorbidity** |  |  |  |  |  |  |
| Hypertension | 47 (18.6) | 73 (35.4) | <0.001 | 32 (27.1) | 41 (46.6) | 0.004 |
| Diabetes | 27 (10.7) | 43 (20.9) | 0.002 | 25 (21.2) | 18 (20.5) | 0.898 |
| Cardiovascular diseases | 8 (3.2) | 43 (20.9) | <0.001 | 25 (21.2) | 18 (20.5) | 0.898 |
| Bronchitis | 17 (6.7) | 17 (8.3) | 0.533 | 6 (5.1) | 11 (12.5) | 0.056 |
| **Blood cell count** |  |  |  |  |  |  |
| WBCs, ×10^9^/L | 6.30 (5.30-7.40) | 7.27 (4.98-11.93) | <0.001 | 7.01 (4.84-10.16) | 8.61 (5.41-13.42) | 0.020 |
| Neutrophils, ×10^9^/L | 4.96 (3.80-6.11) | 5.96 (3.56-10.11) | <0.001 | 5.16 (3.43-7.91) | 7.33 (4.17-13.07) | 0.002 |
| Lymphocytes, ×10^9^/L | 1.46 (1.1-1.845) | 0.77 (0.46-1.14) | <0.001 | 0.82 (0.52-1.29) | 0.68 (0.37-1.00) | 0.008 |
| Monocytes, ×10^9^/L | 0.51 (0.37-0.63) | 0.43 (0.26-0.64) | 0.497 | 0.45 (0.29-0.65) | 0.37 (0.20-0.65) | 0.240 |
| RBCs, ×10^12^/L | 4.27 (3.96-4.72) | 3.46 (2.90-4.08) | <0.001 | 3.48 (2.89-4.06) | 3.40 (2.91-4.17) | 0.697 |
| Hemoglobin, g/L | 136 (121-146) | 107 (86-124) | <0.001 | 108 (88-125) | 106 (83-124) | 0.757 |
| HCT, % | 39.3 (36.1-42.5) | 32.2 (26.6-37.2) | <0.001 | 32.5 (26.5-37.3) | 31.9 (26.7-37.0) | 0.928 |
| Platelets, ×10^9^/L | 239 (189-283) | 175 (107-270) | 0.003 | 199 (141-276) | 138 (84-203) | 0.001 |
| MPV, fL | 10.6 (10.2-11.4) | 10.9 (10.3-12.0) | 0.006 | 10.7 (10.2-11.5) | 11.4 (10.4-12.8) | 0.003 |
| **Coagulation factor** |  |  |  |  |  |  |
| Prothrombin time, s | 12.7 (11.8-13.7) | 13.2 (12.0-15.0) | 0.845 | 12.7 (11.7-13.9) | 14.2 (12.6-16.7) | <0.001 |
| INR | 1.13 (1.04-1.24) | 1.10 (1.00-1.27) | 0.003 | 1.07 (0.99-1.18) | 1.13 (1.02-1.45) | 0.004 |
| aPTT, s | 29.9 (27.8-31.5) | 31.4 (28.2-36.2) | <0.001 | 30.8 (28.0-35.3) | 31.7 (28.6-37.5) | 0.237 |
| Thrombin time, s | 15.5 (14.4-16.6) | 17.3 (16.1-18.4) | <0.001 | 17.1 (16.3-18.2) | 17.6 (15.9-19.0) | 0.324 |
| Fibrinogen, g/L | 3.73 (3.21-4.28) | 4.20 (3.43-5.55) | <0.001 | 4.19 (3.45-5.51) | 4.30 (3.36-5.58) | 0.787 |
| D-dimer, mg/L | 0.47 (0.22-0.75) | 3.17 (1.01-6.24) | <0.001 | 2.20 (0.90-5.27) | 3.67 (1.43-7.22) | 0.017 |
| **Metabolic panel** |  |  |  |  |  |  |
| CRP, mg/L | 22.50 (12.55-54.00) | 29.45 (12.75-69.55) | 0.120 | 26.75 (12.58-62.97) | 33.55 (13.00-72.88) | 0.651 |
| PCT, ng/mL | 1.12 (0.48-1.64) | 0.64 (0.25-1.72) | 0.008 | 0.69 (0.25-1.76) | 0.64 (0.24-1.59) | 0.642 |
| Total bilirubin, μmol/L | 16.50 (11.90-26.05) | 18.50 (13.68-28.73) | 0.017 | 20.50 (15.10-39.15) | 17.55 (9.90-26.10) | 0.009 |
| Direct bilirubin, μmol/L | 6.40 (3.95-12.70) | 8.60 (4.48-16.33) | 0.017 | 9.95 (4.50-21.70) | 7.40 (4.10-11.80) | 0.005 |
| Indirect bilirubin, μmol/L | 9.50 (5.70-13.50) | 10.90 (7.70-17.15) | 0.002 | 11.20 (7.85-18.65) | 10.40 (7.43-15.10) | 0.146 |
| ALT, U/L | 24.20 (18.05-36.30) | 38.65 (30.73-47.35) | <0.001 | 39.00 (33.80-49.00) | 35.75 (24.48-46.73) | 0.049 |
| AST, U/L | 22.70 (14.85-38.75) | 40.75 (30.45-54.35) | <0.001 | 42.60 (33.20-53.98) | 38.20 (27.50-57.20) | 0.226 |
| ALP, U/L | 66.00 (54.00-91.50) | 70.00 (50.00-93.40) | 0.643 | 70.00 (50.00-89.50) | 70.50 (50.00-99.75) | 0.469 |
| GGT, U/L | 48.00 (26.00-71.00) | 45.00 (26.90-73.93) | 0.990 | 45.55 (26.00-71.00) | 43.35 (26.70-67.75) | 0.514 |
| Total protein, g/L | 58.00 (53.10-63.80) | 58.80 (52.78-66.30) | 0.119 | 58.80 (52.78-65.28) | 58.85 (52.53-66.68) | 0.906 |
| Albumin, g/L | 33.50 (29.55-37.40) | 33.00 (29.22-37.73) | 0.592 | 33.10 (29.23-38.10) | 32.85 (29.03-37.43) | 0.847 |
| Globulin, g/L | 24.10 (19.80-28.25) | 25.25 (20.20-29.73) | 0.046 | 24.85 (20.28-29.63) | 25.60 (20.20-30.40) | 0.801 |
| ADA, U/L | 14.30 (11.25-17.55) | 13.60 (10.90-18.73) | 0.731 | 13.60 (10.90-19.90) | 13.60 (11.13-18.38) | 0.838 |
| BUN, mmol/L | 4.40 (3.40-5.50) | 8.55 (4.89-12.35) | <0.001 | 9.05 (4.78-12.90) | 8.15 (5.29-11.65) | 0.621 |
| Creatinine, μmol/L | 61.10 (51.30-74.55) | 69.30 (56.73-86.83) | <0.001 | 69.60 (56.95-85.10) | 68.90 (55.88-93.70) | 0.771 |
| Glucose, mmol/L | 8.75 (6.53-12.40) | 8.44 (5.83-12.98) | 0.498 | 9.34 (5.63-13.63) | 7.95 (6.33-11.72) | 0.185 |
| LDH, U/L | 372.00 (227.00-533.00) | 343.00 (217.50-506.25) | 0.999 | 346.50 (217.00-535.50) | 341.50 (216.75-504.00) | 0.911 |
| CPK, U/L | 62.00 (47.00-90.00) | 72.25 (50.75-138.25) | 0.001 | 70.00 (48.00-136.25) | 82.00 (53.00-153.25) | 0.223 |
| CK-MB, U/L | 55.60 (34.90-76.60) | 30.80 (19.30-60.75) | <0.001 | 32.80 (23.58-63.30) | 26.40 (16.20-54.48) | 0.016 |
| Potassium, mmol/L | 4.19 (3.66-4.59) | 4.02 (3.47-4.33) | <0.001 | 3.98 (3.45-4.31) | 4.06 (3.52-4.33) | 0.394 |
| Sodium, mmol/L | 142.70 (137.00-147.10) | 141.35 (137.48-146.75) | 0.828 | 143.70 (138.25-146.95) | 139.50 (137.00-145.80) | 0.064 |

Abbreviations: WBC, white blood cell; RBC, red blood cell; HCT, hematocrit; MPV, mean platelet volume; INR, international normalized ratio; aPTT, activated partial thromboplastin time; CRP, c-reactive protein; PCT, procalcitonin; ALT, alanine aminotransferase; AST, aspartate aminotransferase; ALP, alkaline phosphatase; GGT, γ-glutamyl transferase; ADA, adenosine deaminase; BUN, blood urea nitrogen; LDH, lactate dehydrogenase; CPK, creatine phosphokinase; CK-MB, creatine kinase isoenzyme.

**Supplementary Table 2.** Multivariate analysis of independent risk factors to predict COVID-19 severity.

| **Variables** | ***p*** | **Odds Ratio** | **95% Confidence Interval** |
| --- | --- | --- | --- |
| Age (years) | <0.001 | 1.036 | (1.016,1.057) |
| Male (%) | 0.973 | 1.012 | (0.507,2.020) |
| Hypertension | 0.026 | 3.007 | (1.140,7.935) |
| Diabetes | 0.434 | 0.655 | (0.226,1.893) |
| WBCs, ×10^9^/L | 0.530 | 1.060 | (0.883,1.273) |
| Neutrophils, ×10^9^/L | 0.515 | 1.058 | (0.894,1.251) |
| Lymphocytes, ×10^9^/L | 0.057 | 0.662 | (0.432,1.013) |
| RBCs, ×10^12^/L | <0.001 | 0.425 | (0.291,0.622) |
| ALT, U/L | 0.032 | 1.014 | (1.001,1.027) |
| AST, U/L | 0.686 | 1.002 | (0.994,1.009) |
| BUN, mmol/L | <0.001 | 1.257 | (1.152,1.373) |
| Creatinine, μmol/L | 0.637 | 1.002 | (0.995,1.009) |
| Potassium, mmol/L | <0.001 | 0.404 | (0.244,0.668) |
| D-dimer, mg/L | <0.001 | 1.392 | (1.193,1.623) |

**Supplementary Table 3.** Multivariate analysis of independent risk factors to predict COVID-19 mortality.

| **Variables** | ***p*** | **Odds Ratio** | **95% Confidence Interval** |
| --- | --- | --- | --- |
| Hypertension | 0.021 | 2.097 | (1.116,3.939) |
| WBCs, ×10^9^/L | 0.827 | 0.983 | (0.846,1.143) |
| Neutrophils, ×10^9^/L | 0.330 | 1.077 | (0.927,1.251) |
| Lymphocytes, ×10^9^/L | 0.214 | 0.784 | (0.535,1.150) |
| Platelet, ×10^9^/L | 0.174 | 0.998 | (0.995,1.001) |
| MPV, fL | 0.188 | 1.158 | (0.931,1.441) |
| Prothrombin time, s | 0.806 | 1.019 | (0.877,1.185) |
| INR | 0.532 | 1.505 | (0.418,5.420) |
| D-dimer, mg/L | 0.430 | 1.012 | (0.982,1.043) |
